# *Lactiplantibacillus plantarum* Lp05 protects against ethanol-induced liver injury in zebrafish through metabolic and microbiota modulation

**DOI:** 10.1038/s41598-025-07111-5

**Published:** 2025-07-02

**Authors:** Yixuan Fan, Yao Dong, Zhonghui Gai, Yanyan Zhang, Mei Han

**Affiliations:** 1 Department of Food Quality and Safety, Shanghai Business School, Shanghai, 200235 China; 2 Department of Research and Development, Wecare Probiotics Co., Ltd, Suzhou, 215200 China; 3 Germline Stem Cells and Microenvironment Lab, College of Animal Science and Technology, Nanjing Agricultural University, Nanjing, 210095 China

**Keywords:** *Lactiplantibacillus plantarum*, Ethanol-induced liver injury, Metabolic pathways, 16S rRNA sequencing, Zebrafish model, Bacterial host response, Metagenomics

## Abstract

This study investigates the protective effects of *Lactiplantibacillus plantarum* Lp05 on ethanol-induced liver injury in zebrafish (*Danio rerio*), focusing on its mechanisms related to metabolic pathway regulation and gut microbiota composition. We administered Lp05 to zebrafish exposed to ethanol to assess its impact on liver enzymes and overall liver health. Key metrics included the expression levels of alcohol dehydrogenase (ADH) and aldehyde dehydrogenase (ALDH), activity of serum alanine aminotransferase (ALT) and aspartate aminotransferase (AST), and changes in gut microbiota composition through 16S rRNA sequencing. Metabolomic analyses were conducted to identify affected metabolic pathways. Treatment with Lp05 significantly increased ADH and ALDH expression, enhancing alcohol metabolism and reducing ALT and AST activities, thereby mitigating liver cell damage. Metabolomic analysis revealed significant modulation of biotin and glycerolipid metabolism pathways, crucial for reducing liver injury. Lp05 also altered gut microbiota, increasing beneficial bacteria such as *Fluviicola* and *Delftia*, while decreasing pathogenic bacteria like *Acinetobacter* and *Aeromonas*. This bacterial modulation contributed to phenylalanine metabolism regulation, which alleviated intestinal inflammation and liver injury. Additionally, Lp05 promoted the degradation of polycyclic aromatic hydrocarbons (PAHs), reducing their health risks. Lp05 exhibits potential therapeutic effects against ethanol-induced liver injury in zebrafish by enhancing alcohol metabolism, modulating metabolic pathways, and altering gut microbiota. These findings suggest that Lp05 may offer a novel preventative and therapeutic strategy for managing alcoholic liver injury. Alcohol-induced liver injury is a significant global health concern with limited effective therapeutic options. This study highlights the potential of *Lactiplantibacillus plantarum* Lp05 as a novel probiotic intervention for mitigating ethanol-induced liver damage. By enhancing alcohol metabolism, regulating critical metabolic pathways such as biotin and glycerolipid metabolism, and modulating gut microbiota composition, Lp05 addresses both the metabolic and microbiological aspects of liver health. The promotion of beneficial bacteria and the suppression of pathogenic strains further contribute to alleviating liver injury and systemic inflammation. These findings underscore the therapeutic promise of Lp05 in managing alcoholic liver injury and provide a foundation for its development as a preventative and therapeutic strategy in human applications.

## Introduction

The liver, a vital organ in humans, plays a critical role in maintaining overall health through its involvement in detoxification, metabolism, and blood regulation. Due to these functions, the liver is highly susceptible to damage from exogenous chemicals, leading to various liver diseases. Chemical liver injuries, triggered by agents such as pharmaceuticals, alcohol, industrial solvents, and heavy metals, can evolve into severe conditions like liver fibrosis and cirrhosis^1,2^. In recent years, the rising prevalence of alcohol consumption has notably increased the incidence of liver injury^3^. Alcohol metabolism generates toxic byproducts such as acetaldehyde and reactive oxygen species (ROS), which are detrimental to hepatocytes. These substances induce oxidative stress and inflammatory responses, promoting lipid peroxidation and subsequent cell membrane damage, culminating in hepatocyte injury and necrosis^4,5^. Additionally, alcohol intake exacerbates gut dysbiosis, increasing the proliferation of pathogenic bacteria and enhancing endotoxin production, which further aggravates liver damage^6^. While mild chemical liver injuries induced by alcohol may be reversible through abstinence, advanced liver disease often necessitates living-donor liver transplantation as a therapeutic strategy. However, the outcomes of transplantation are not always favorable, complicated by donor organ shortages and the challenges of immune rejection^7^. Metadoxine, a well-known hepatoprotective agent, alleviates alcohol-induced liver damage by promoting fatty acid metabolism, reducing lipid accumulation in hepatocytes, and safeguarding liver function. However, it has certain drawbacks, including potential side effects such as gastrointestinal discomfort and rashes, as well as varying efficacy depending on individual differences^8^. Given these limitations, there is a growing interest in exploring alternative therapies that can prevent progression or even reverse the damage at earlier stages.

Probiotics, defined as live microorganisms that confer health benefits when consumed in adequate amounts, have demonstrated promising therapeutic potential in liver health management. Through the gut-liver axis, probiotics enhance intestinal barriers and reduce the translocation of toxic substances, thereby mitigating the impact of harmful chemicals on the liver^9,10^. Nanji et al. first highlighted the potential of probiotics in supporting liver health in 1994, finding that the *Lactobacillus* GG strain (LGG) could reduce liver damage in rats administered alcohol^11^. In 2006, Ewaschuk et al. further confirmed that LGG strengthens intestinal barrier functions and the mucosal immune system, producing broad-spectrum antimicrobial substances such as bacteriocins and hydrogen peroxide. These substances significantly reduce the prevalence of alcohol-induced Gram-negative bacteria and the production of endotoxins in the gut, which leads to increased levels of endotoxins in the blood and liver, resulting in fat accumulation and inflammatory responses^11–15^. Wang et al.’s research also showed that LGG helps alleviate liver injury by upregulating the expression of genes that protect the intestinal mucosal barrier, such as ITF and tight junction proteins, thus preventing endotoxin leakage^16,17^. Additionally, research by Dong and Li has found that the *Bifidobacterium longum* subspecies BL21 and *Lactobacillus plantarum* J26 significantly improve alcohol-related liver disease by enhancing liver antioxidative capabilities and regulating the gut microbiome^18,19^. However, existing studies have not yet thoroughly explored the mechanisms by which probiotics intervene in chemical liver injury.

Therefore, this study aims to investigate the effects of *Lactiplantibacillus plantarum* Lp05 on ethanol-induced chemical liver injury in a zebrafish model and its underlying mechanisms. Due to its highly conserved genetic background and similarity to human disease models, the zebrafish serves as an ideal model for studying human diseases. By integrating 16 S rRNA sequencing with metabolomics analysis, we aim to comprehensively understand the role of probiotics in chemical liver injury, providing new strategies for the prevention and treatment of this condition.

## Results

### Assessment of auxiliary protective efficacy

**Fig. 1:**
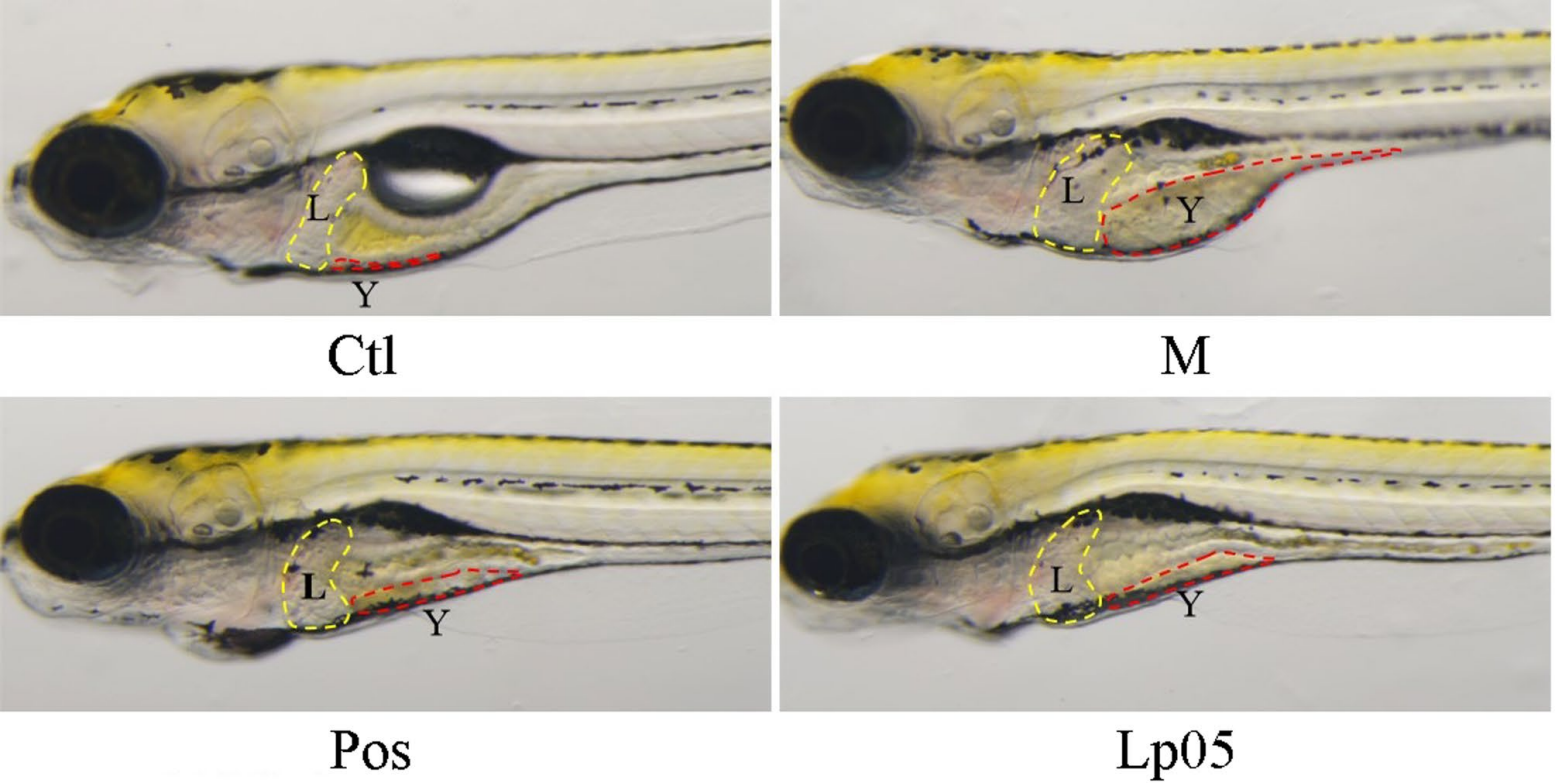
Zebrafish liver and yolk sac area. The L and yellow dashed lines represent the liver; the Y and red dashed lines represent the yolk sac. Ctl, the control group; M, model group; Pos, positive control group; Lp05, *Lactiplantibacillus plantarum* Lp05.

**Fig. 2:**
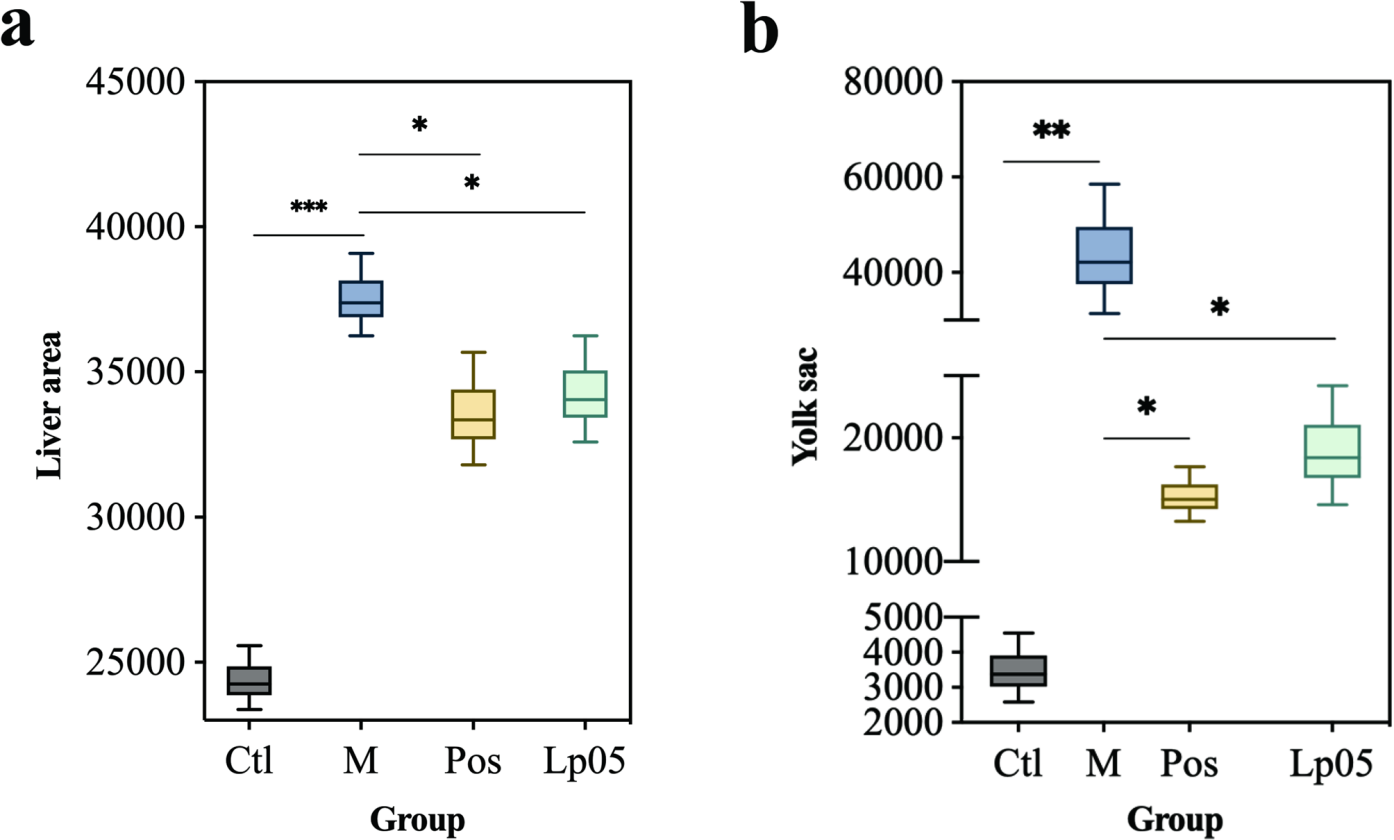
Zebrafish liver and yolk sac areas. (a) zebrafish liver area; (b) zebrafish yolk sac area. * *p* value < 0.05; ** *p* value < 0.01; *** *p* value < 0.001. Ctl, the control group; M, model group; Pos, positive control group; Lp05, *Lactiplantibacillus plantarum* Lp05.

As shown in Fig. 1, the liver area (indicated by the yellow dashed box) and yolk sac area (indicated by the red dashed box) were significantly enlarged in the model (M) group compared to the control (Ctl) group. In contrast, treatment with metadoxine (Pos group) and Lp05 (Lp05 group) markedly reduced the enlargement of both the liver and yolk sac areas relative to the model group, indicating a protective effect against ethanol-induced hepatomegaly and yolk sac edema. In contrast, the liver and yolk sac areas of the zebrafish treated with Lp05 (3 × 10^6^ CFU/mL) were essentially similar to those of the Pos group treated with metadoxine) (Fig. 2a and b).

**Fig. 3:**
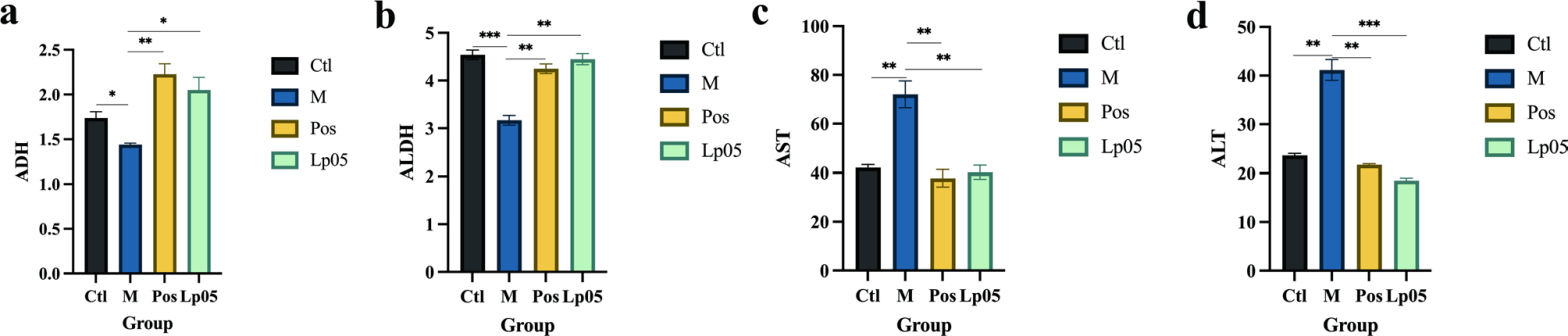
ADH, ALDH, AST and ALT levels in zebrafish. (a) ADH; (b) ALDH; (c) AST; (d) ALT. * *p* value < 0.05; ** *p* value < 0.01; *** *p* value < 0.001. Ctl, the control group; M, model group; Pos, positive control group; Lp05, *Lactiplantibacillus plantarum* Lp05.

The results of ADH and ALDH area in zebrafish revealed significant differences among the groups. Specifically, the M group exhibited markedly lower ADH levels compared to the other groups (Fig. 3a). Notably, the Pos group treated with metadoxine displayed the highest ADH levels. A similar pattern was observed for ALDH levels, with the M group showing the lowest ALDH levels (Fig. 3b). Additionally, the ALDH levels in the Lp05 treatment group were found to be higher than those in the Pos group.

After exposure to ethanol stimulation, the activity of ALT in the M group significantly increased, accompanied by an enhancement in the activity of AST. However, after treatment with metadoxine and Lp05, the activities of both AST and ALT showed a significant decrease, with the Lp05 group maintaining at a lower level (Fig. 3c and d).

### Liver histopathological sections

**Fig. 4:**
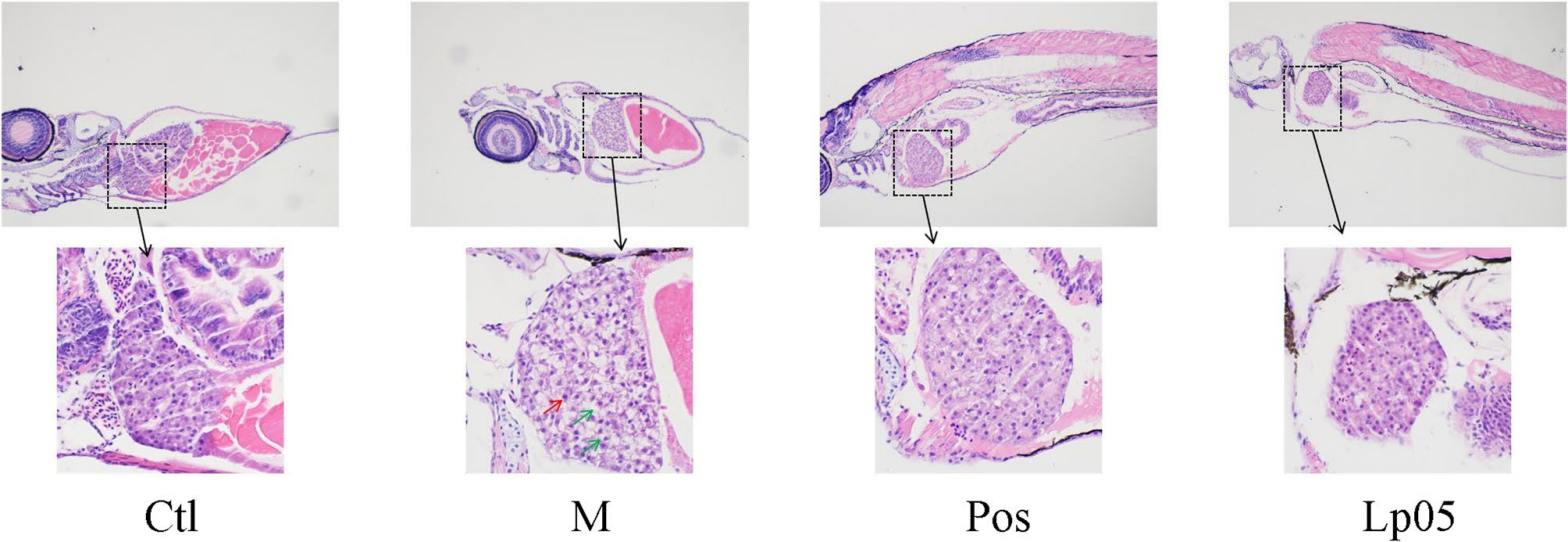
Hiopathological section of zebrafish liver. Red arrows in the figure represent enlarged hepatocyte nucleus, and green arrows represent fatty vacuolar-like degeneration. Ctl, the control group; M, model group; Pos, positive control group; Lp05, *Lactiplantibacillus plantarum* Lp05.

Histopathological examination of liver tissue sections revealed that in the control (Ctl) group, hepatocytes displayed regular morphology, distinct nuclei, and well-defined cellular boundaries. In contrast, the model (M) group exhibited disorganized hepatic architecture, nuclear enlargement (indicated by red arrows), and pronounced fatty vacuolar degeneration (indicated by green arrows), confirming the successful establishment of ethanol-induced liver injury. Notably, the livers of the Pos and Lp05 groups showed clearer cellular structures, reduced lipid vacuole formation, and significant improvement in histopathological features compared to the model group (Fig. 4).

### Liver ultrastructure

**Fig. 5:**
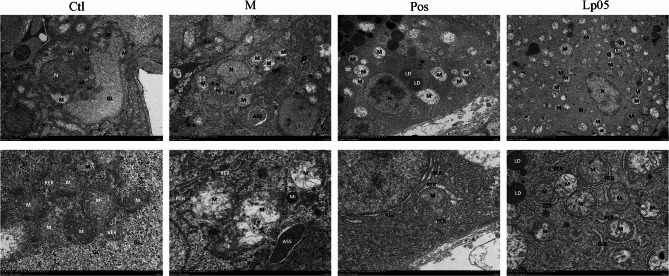
Zebrafish liver ultragraph. (N) nucleus; (M) mitochondria; (RER) rough endoplasmic reticulum; (GL) glycogen; (Mi) microbodies; (ASS) autophagosomes; (LD) lipid droplets. Ctl, the control group; M, model group; Pos, positive control group; Lp05, *Lactiplantibacillus plantarum* Lp05.

The ultrastructure of zebrafish livers revealed distinct cellular damage in the M group, characterized by a scarcity of organelles with predominant mitochondrial damage. Mitochondria appeared swollen and enlarged with disrupted membranes, matrix dissolution, and effusion. There was substantial dissolution of cristae and observable autophagy. The Pos group exhibited relatively less cellular damage, also with a scarcity of organelles and predominant mitochondrial injury. Most mitochondria were swollen and enlarged, with intact membranes, matrix dissolution, vacuolation, and extensive dissolution of cristae. The Ctl group and the Lp05 group showed relatively mild cellular damage, with visible lipid droplets and slight fusion. The former had fewer organelles, abundant glycogen, and most organelles maintained their structure despite some mitochondria being swollen. Lp05 also had abundant organelles, with most showing matrix dissolution and reduced cristae, and a minority with severe damage exhibiting membrane rupture (Fig. 5).

### Metabolomic analysis

**Fig. 6:**
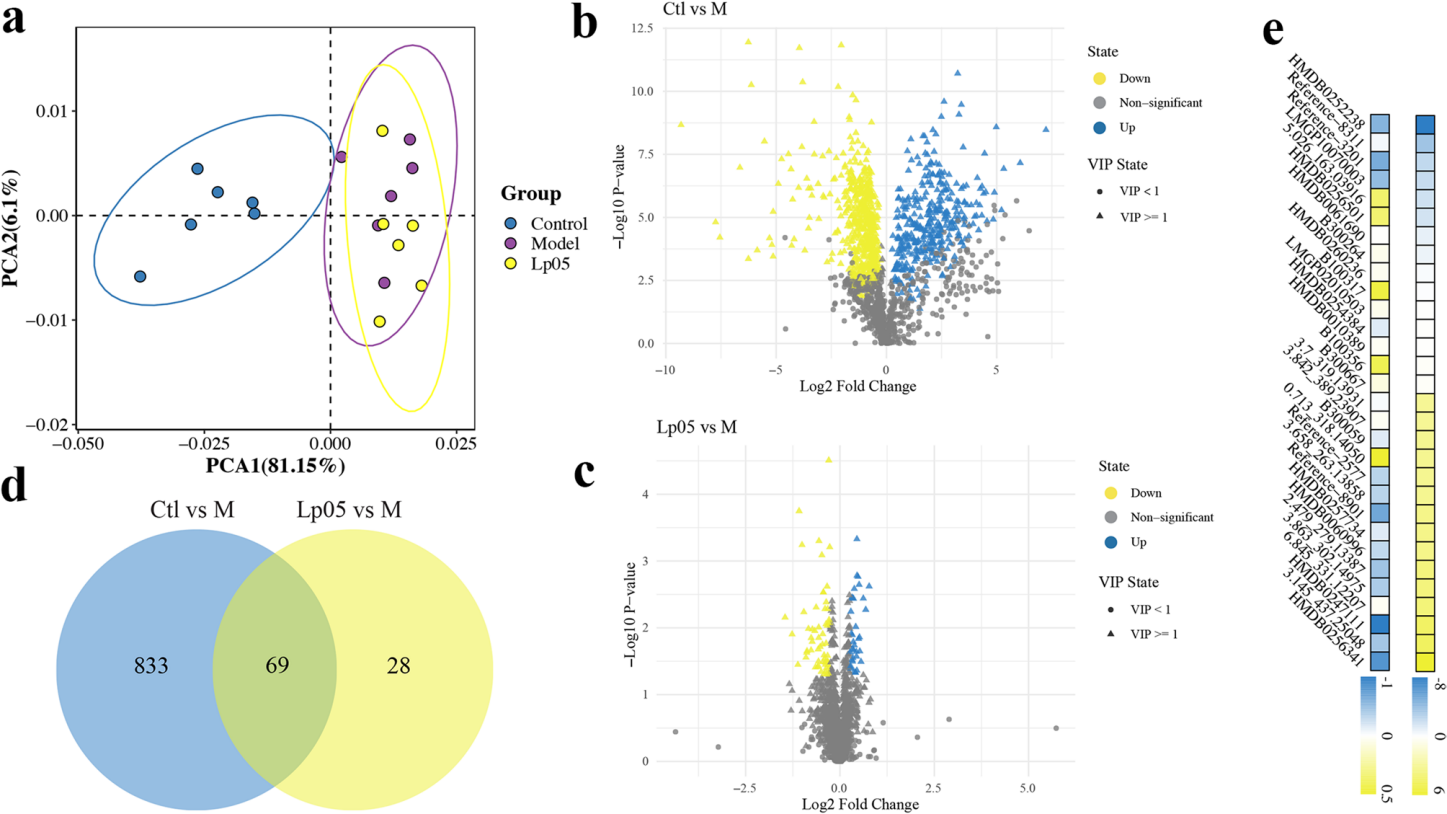
Zebrafish Metabolic Figures. (a) PCA plot; (b) volcano plot of differential metabolites for Ctl and M; (c) volcano plot of differential metabolites for Lp05 and M; (d) venn diagram of differential metabolites between Ctl vs. M and Lp05 vs. M; (e) heatmap of the top 30 most differential metabolites of 69 differential metabolites. Ctl, the control group; M, model group; Lp05, *Lactiplantibacillus plantarum* Lp05.

Principal Component Analysis (PCA) indicated a clear separation trend between the Ctl group and the M group, suggesting significant differences in metabolites between the two groups; while there was an overlapping area between the Lp05 and M groups, indicated that a large number of metabolites are common between the two groups (Fig. 6a).

By setting the VIP ≥ 1, Fold Change ≥ 1.2 or ≤ 0.83, and p-value < 0.05 for the Orthogonal Partial Least Squares-Discriminant Analysis (OPLS-DA) model, the results showed that, there were a total of 358 upregulated differential metabolites in the Ctl group and 544 downregulated differential metabolites compared to the M group; while Lp05 had a total of 36 upregulated and 61 downregulated differential metabolites compared to the M group (Fig. 6b and c). A comparison of the shared and unique differential metabolites between revealed that there are 69 shared differential metabolites between Ctl vs. M and Lp05 vs. M (Fig. 6d). These 69 metabolites were sorted by Log2FC values, and the top 15 upregulated and top 15 downregulated metabolites are highlighted (Fig. 6e).

**Fig. 7:**
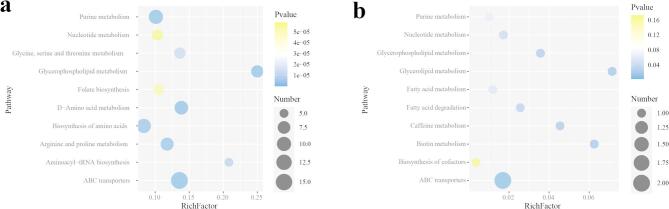
KEGG Pathway Analysis of Differential Metabolites. (a) pathway map of differential metabolites for Ctl vs. M; (b) pathway map of differential metabolites for Lp05 vs. M. The size of the circles in the graph represents the number of differential metabolites contained in that pathway; the larger the circle, the more differential metabolites it contains. The bluer the circle color, more significant the pathway. Ctl, the control group; M, model group; Lp05, *Lactiplantibacillus plantarum* Lp05.

In-depth analysis of differential metabolites revealed that the two most significantly enriched KEGG metabolic pathways between Ctl vs. M and Lp05 vs. M were the ABC transporter pathway and the glycerophospholipid metabolism pathway (Fig. 7a and b). Furthermore, treatment with Lp05 induced significant alterations in the Biotin metabolism and Glycerolipid metabolism pathways (Fig. 7b).

### The 16 S rRNA sequencing

**Fig. 8:**
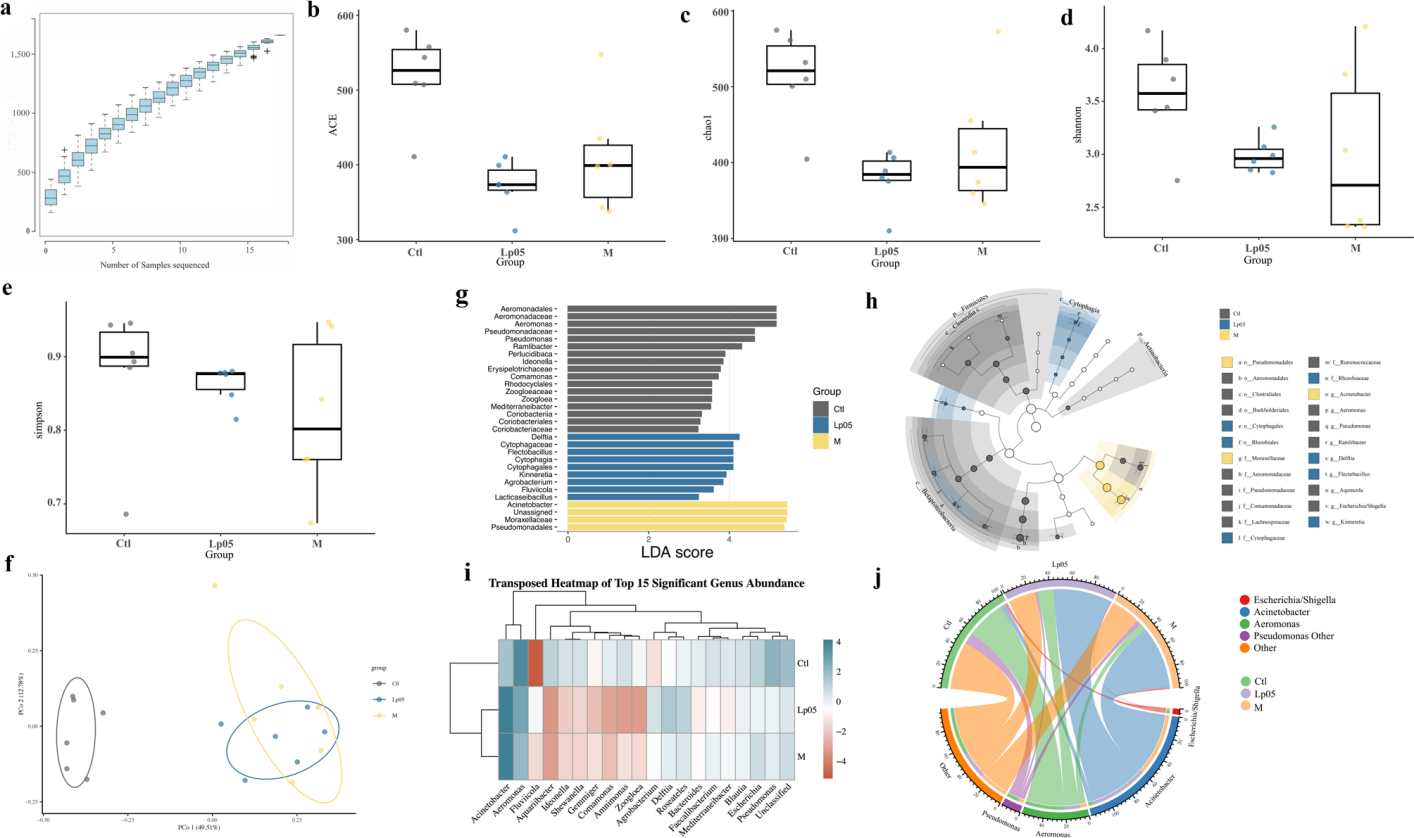
The 16S rRNA sequencing results. (a) species accumulation curve; (b) ACE; (c) Chao1; (d) Shannon; (e) Simpson; (f) LEfSe bae chart; (g) LEfSe cladogram; (h) Pcoa of Beta analysis; (i) heatmap of top 15 significant abundance genus; (j) circos plot of the five most abundant genus. Ctl, the control group; M, model group; Lp05, *Lactiplantibacillus plantarum* Lp05.

The species accumulation curve from 16 S rRNA sequencing result indicated that a leveling off in the upward trend at its terminal end, suggesting that the sample volume for this sequencing was sufficient (Fig. 8a). The indices of ACE and chao1 revealed that the Ctl group possessed the most diverse microbial community, while the Lp05 group exhibited a slightly lower richness compared to the M group (Fig. 8b and c). In terms of community diversity, the Ctl group demonstrated higher diversity than both the Lp05 and M groups and the Lp05 group showed a marginally greater diversity than the M group (Fig. 8d and e). Beta diversity analysis indicateed minor differences between the communities of the Lp05 and M groups, yet both exhibited a significantly higher diversity compared to the Ctl group (Fig. 8f).

The results from the Linear Discriminant Analysis Effect Size (LEFSe) indicated that the genus *Delftia* had the most significant impact within the Lp05 group (Fig. 8g), and it also overlaped with the region in the Ctl group (Fig. 8h). In addition to *Delftia*, the family *Cytophagaceae*, the genus *Flectobacillus*, and the order *Cytophagales* are also identified as playing crucial roles in the Lp05 group (Fig. 8g and h).

The results depicted in Fig. 8I revealed that among the 15 most significantly differentially abundant genera, the abundance of *Aquariibacter*, *Ideonella*, *Shewanella*, *Gemmiger*, *Comamonas*, *Amnimonas*, and *Zoogloea* significantly decreased in both the Lp05 and M groups, and *Comamonas*, *Amnimonas*, and *Zoogloea* showed even lower abundance in the Lp05 group compared to the M group. In contrast, the abundance of *Fluviicola* and *Delftia* was highest in the Lp05 group.

The circos plot of the five most abundant genus showed that *Escherichia/Shigella* was annotated exclusively in the Lp05 and Ctl groups; *Acinetobacter* was annotated only in the Lp05 and M groups. In addition, both *Pseudomonas* and *Aeromonas* were annotated across all three groups, but with a higher number of annotations in the Ctl group (Fig. 8j).

**Fig. 9:**
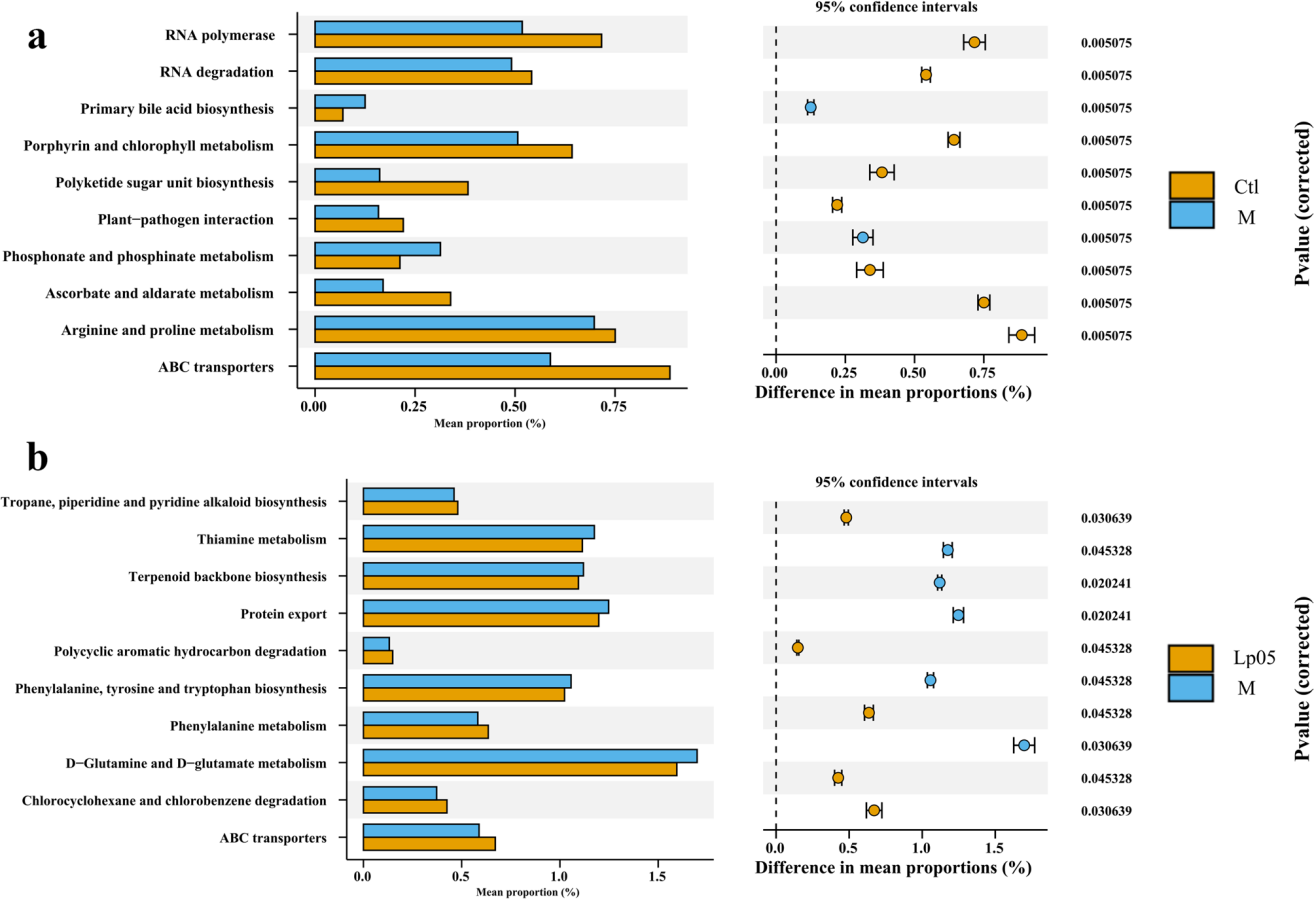
Stamp analysis of Ctl vs. M and Lp05 vs. M. (a) stamp result of Ctl vs. M; (b) stamp result of Lp05 vs. M. Ctl, control group; M, model group; Lp05, *Lactiplantibacillus plantarum* Lp05.

The KEGG annotation results from PICRUST2 the 10 most significantly differential pathways between Ctl vs. M and Lp05 vs. M. ABC transporters pathway was a common differential metabolic pathway between these two. Compared to the Ctl group, the primary metabolic pathways in the M group were focused on several metabolic pathways such as Ascorbate and aldarate metabolism, suggesting that alcohol might induce a decrease in the expression of these pathways in the tested zebrafish, and stimulate the high expression of Primary bile acid biosynthesis and Phosphonate and phosphinate metabolism (Fig. 9a). While in Lp05 group, several metabolic pathways exhibited heightened expression included Tropane, piperidine and pyridine alkaloid biosynthesis, Polycyclic aromatic hydrocarbon degradation, Phenylalanine metabolism, and Chlorocyclohexane and chlorobenzene degradation showed higher expression levels in the Lp05 group. While the expression of Thiamine metabolism, Terpenoid backbone biosynthesis, Protein export, Phenylalanine, tyrosine and tryptophan biosynthesis, and D − Glutamine and D − glutamate metabolism demonstrated increased in the M group (Fig. 9b).

## Discussion

Alcohol-induced liver injury has been extensively studied using zebrafish models, which exhibit histopathological and ultrastructural changes such as hepatic steatosis and inflammation following ethanol exposure^20–22^. In recent years, the indispensable role of probiotics in maintaining intestinal and liver health has gained increasing recognition^20–22^. Probiotics have also been demonstrated to alleviate symptoms in animal models of alcoholic liver disease^16,17^. Based on this evidence, this study utilized *Lactiplantibacillus plantarum* Lp05 as the research subject to evaluate its protective effects against ethanol-induced liver injury in zebrafish. By integrating metabolomic and 16S rRNA sequencing data, we further explored the interactions between gut microbiota, differential metabolites, and liver injury, as well as the potential protective mechanisms.

Previous studies have indicated that low expression of ADH and ALDH is directly associated with poor prognosis in Hepatocellular Carcinoma (HCC)^23,24^ and their high expression can promote the metabolism of alcohol, alleviating liver injury caused by excessive alcohol levels^24^. ALT is primarily found in the liver, while AST is predominantly present in the heart and liver^25^. Elevated activities of AST and ALT suggest damage to liver or heart muscle cells, leading to increased serum levels of these enzymes^26,27^. In ethanol-induced liver injury, the downregulation of these two enzymes leads to the accumulation of ethanol and its toxic intermediate, acetaldehyde, triggering oxidative stress and cellular damage. Experimental results indicate that Lp05 promotes efficient metabolism and clearance of ethanol by upregulating the expression of these two key enzymes (ADH/ALDH), thereby alleviating the toxic burden on hepatocytes. This regulatory mechanism not only explains the improvement of Lp05 in liver function markers (ALT/AST), but also aligns with its protective effects on hepatic histology and ultrastructure. Notably, compared to the positive control group (metadoxine), the Lp05 treatment group achieved similar enzyme activity regulation effects, but Lp05 exhibited more pronounced protection of hepatocyte ultrastructure^28–31^ suggesting that Lp05 has a more distinctive cellular protective mechanism.

The metabolomic results indicated that, compared to the Model group, significant changes occurred in Biotin metabolism and Glycerolipid metabolism in the Lp05 group. The liver is the primary site for the synthesis and metabolism of triglycerides, capable of synthesizing triglycerides and storing them in adipocytes, and also be able to break down triglycerides into glycerol and fatty acids; when triglyceride metabolism is abnormal, it may lead to excessive accumulation of triglycerides in the liver, forming fatty liver, thereby causing structural damage to hepatocytes and affecting their function^32^. Therefore, Lp05 may alleviate hepatic steatosis by regulating the triglyceride metabolism pathway. At the same time, biotin deficiency or metabolic abnormalities have been shown to be associated with various liver diseases^33,34^. It is speculated that Lp05 may promote hepatic homeostasis by optimizing biotin metabolism.

Moreover, the most enriched differential metabolites were associated with ABC transporters. ABC transporters, as highly conserved transmembrane proteins, play a crucial role in the liver detoxification process^35,36^. Compared to the control group, the Lp05 treatment group showed more pronounced changes in ABC transporter-related metabolites in the model group, suggesting that Lp05 may enhance liver detoxification system function and promote the clearance of ethanol and its metabolites.

An important finding of this study is the regulatory effect of Lp05 on the structure and function of the gut microbiome. Although Lp05 treatment did not significantly alter the α and β diversity of gut microbiota, LEFSe analysis and differential heatmaps at the genus level revealed that the abundance of *Fluviicola* and *Delftia* was highest in the Lp05 group, especially the high abundance of *Delftia* is closely associated with phenylalanine metabolism^37^. More importantly, STAMP analysis showed that the biosynthesis of phenylalanine was more prominent in the model group, whereas its metabolism was more active in the Lp05 group. The accumulation of phenylalanine has been shown in previous studies to contribute to liver damage and intestinal barrier dysfunction^38,39^. These results collectively point to a potential protective mechanism: Lp05 may enhance the metabolism of phenylalanine by regulating the abundance of *Delftia*, thereby alleviating liver damage induced by high levels of phenylalanine. Additionally, we found that Lp05 reduced the proliferation of harmful bacteria such as *Acinetobacter* and *Aeromonas*, both of which have been reported to be associated with various diseases, including liver and intestinal infections. *Acinetobacter* can produce endotoxins, triggering inflammatory responses and exacerbating liver injury^40^ while *Aeromonas* can disrupt intestinal barrier integrity, increase intestinal permeability, and promote bacterial translocation and endotoxemia^41^. The inhibitory effect of Lp05 on these harmful bacteria may represent another key mechanism of its hepatoprotective effects, further supporting the critical role of the ‘gut-liver axis’ in the treatment of alcoholic liver disease.

Overall, this study integrated multi-omics analyses to reveal the protective effects and molecular mechanisms of *Lactiplantibacillus plantarum* Lp05 in ethanol-induced liver injury. Lp05 not only promotes ethanol clearance by upregulating the alcohol-metabolizing enzyme system, but also exerts a comprehensive protective effect through the modulation of metabolic networks and regulation of gut microbiome structure. This provides new insights into the potential of probiotics as adjunctive therapy for alcoholic liver disease. However, the study did not explore the relationship between Lp05 dosage and its protective effects on liver injury, and the use of zebrafish models may not fully replicate human physiology. Nevertheless, future studies could systematically evaluate different doses of Lp05, combined with metagenomics or metatranscriptomics, in rodent or primate models, to further advance the clinical application of Lp05 in alcoholic and other metabolic liver diseases.

## Materials and methods

### Experimental materials

Wild-type AB strain zebrafish (Huantie Biology, China) were used as experimental animals. Main equipment included a dissecting microscope (OLYMPUS SZX7, Japan), a microplate reader (TECAN SPARK, Austria), a transmission electron microscope (Hitachi HT7800/HT7700, Japan), a microtome (KD2258, China), a refrigerated centrifuge (ThermoFisher Heraeus Fresco17, Germany), a thermostatic water bath (HHS-2 S, China), and a heating plate (400 × 280 mm, China). Key reagents included metadoxine, DMSO, anhydrous ethanol, ELISA kits for ADH and ALDH, GPT/ALT and GOT/AST assay kits, 4% tissue fixative, xylene, Mayer’s hematoxylin, eosin staining solution, neutral gum, and paraffin for sectioning (all sourced from certified suppliers in China). All reagents were of analytical grade and used following the manufacturers’ instructions.

### Preparation of strain Lp05

Under sterile conditions, the *Lactobacillus plantarum* Lp05 strain was handled and cultured. This strain was provided by Wecare Probiotics Co. Ltd. The culture was grown in modified MRS medium supplemented with 0.1% L-cysteine hydrochloride to optimize growth conditions. The strain was incubated at 37 °C in an anaerobic environment for 22 to 24 h, after which the cells were harvested by centrifugation (6000*×g* for 8 min at 4 °C)^42^. The harvested cells were resuspended in sterile water, adjusted to a concentration of 3 × 10^6^ CFU/mL, and stored at 4 °C for subsequent use.

### Laboratory animal

Wild-type AB strain zebrafish were randomly maintained in six-well plates containing standard water at a temperature of 28 °C. By adding 200 mg of instant sea salt to every 1 L of reverse osmosis water to afford the standard water with the conductivity from 450 to 550 µS/cm, pH ranges from 6.5 to 8.5 and a hardness from 50 to 100 mg/L CaCO_3_. Wild-type AB strain zebrafish at 3 days post-fertilization (3 dpf) were randomly assigned to four groups (90 fish per group, evenly distributed across six-well plates): Control (Ctl), Alcohol model (M), Lp05 treatment (Lp05), and Positive control (Pos).

### Experimental design

Except for the control group, the remaining groups were exposed to an aqueous solution of anhydrous ethanol (1.67% v/v) to establish a zebrafish liver injury model. In the Lp05 treatment group, Lp05 solution was added at a dose of 3 × 10^6^ CFU/mL. Metadoxine, known for its significant hepatoprotective effects, was chosen as the positive control (Pos) group, with metadoxine dissolved in DMSO to prepare a 149 µg/mL solution. The Pos group received the metadoxine solution at a concentration of 149 µg/mL. Each well contained 3 mL of solution. The fish were maintained at 28 °C, with daily water changes.

### Protective effect on the liver-phenotype

After two days, ten zebrafish were randomly selected from each group. These zebrafish were then placed under a dissecting microscope for photography. Subsequently, images were analyzed using the NIS-Elements D software (Version: 3.20) to measure and collect data on the liver area and yolk sac area of the zebrafish.

### Protective effect on the liver-biochemical indicators

Thirty whole zebrafish were randomly collected from each group and divided into three biological replicates (ten fish per replicate). Samples were gently washed with ultrapure water to remove surface residues, placed into 1.5 mL EP tubes, and homogenized in 0.9% normal saline using sterile homogenizers. The homogenates were centrifuged at 4 °C, 5000*×g* for 10 min, and the supernatants were carefully collected. The enzyme activities of aspartate aminotransferase (AST), alanine aminotransferase (ALT), alcohol dehydrogenase (ADH), and aldehyde dehydrogenase (ALDH) were measured using commercial assay kits according to the manufacturers’ protocols. Measurements were performed using a multifunctional microplate reader (SPARK, TECAN, Austria), and enzyme activities were calculated based on standard curves generated with known standards^43^.

### Zebrafish liver histopathological sections

Whole zebrafish were fixed in 4% tissue fixative. After fixation, samples were dehydrated, embedded in paraffin, sectioned, and stained with hematoxylin and eosin (H&E) for histopathological analysis. Liver tissue was identified based on zebrafish anatomical structure during microscopic observation to evaluate changes across different groups^44^.

### Liver ultrastructure

Zebrafish samples were fixed with electron microscopy fixative. The samples were first placed in 0.1 M phosphate buffer (PB, pH 7.4) for fixation, followed by dehydration through a series of graded acetone solutions at room temperature. After dehydration, the samples were infiltrated with a mixture of acetone and 812 embedding agent (acetone: 812 agents = 1:1) and subsequently embedded in pure epoxy resin. The blocks were polymerized at 60 °C to form hardened specimens. The specimens were then sectioned into approximately 60–80 nm thick ultrathin slices and placed on copper grids. The sections were stained with uranyl acetate and lead citrate and examined under a transmission electron microscope to observe the hepatic ultrastructure.

### Metabolomics

Every 90 zebrafish were transferred into 1.5 mL EP tubes, the liquid was aspirated^45^ two small magnetic beads, 10 µL of internal standard, and 800 µL of precipitation reagent (methanol: acetonitrile: water, 2:2:1) were added to the samples. After thorough grinding, the mixture was centrifuged at 25,000 g at 4 °C for 15 min. The supernatant (600 µL) was carefully collected and frozen for solvent evaporation. Then, 600 µL of 50% methanol was added, and the mixture was shaken to dissolve. After a second centrifugation, the supernatant was transferred to a new EP tube. Non-targeted metabolomic analysis was conducted using a Waters 2777 C UPLC system coupled with a Q Exactive HF mass spectrometer^46–49^. The result data were imported into Compound Discoverer 3.3 software (Thermo Fisher Scientific, USA) for in-depth analysis combined with the BGI Metabolome Database, mzCloud, and ChemSpider databases to identify and compare to reveal the metabolic changes triggered by experimental conditions, such as liver injury or treatment. This approach was intended to identify potential metabolic biomarkers and provides deeper insights into the underlying biological mechanisms^50^.

### The 16S rRNA sequencing

The 16S rRNA sequencing was used to analyze the microbial community structure in ethanol-induced liver injury zebrafish, enabling precise identification and quantification of microbial species while revealing the diversity and composition of the microbiota in this model. For the 16S rRNA analysis, 30 zebrafish per group were collected, with whole fish samples used. A total of 6 biological replicates were included. 30 ng of qualified genomic DNA were extracted with high-quality along with the corresponding primers to complete the PCR amplification of the target sequence. These PCR products were purified using Agencourt AMPure XP magnetic beads and resuspended in Elution Buffer for the library construction and sequencing. Cutadapt (Version 2.6) was used to filter low-quality regions from the raw data^51^. Unoise3 was utilized for noise reduction to achieve single-base precision amplicon sequence variants (ASVs) and an operational taxonomic unit (OTU) characteristic table was generated. The alpha and beta diversities were analyzed using Usearch (Version 10.0), PICRUSt2 (Version 2.5.2) was employed to predict the functional profiles based on the KEGG database using the ASV table generated from the sequencing data^52–55^. Subsequently, the corresponding figures were plotted using R (Version 4.3)^50^.

### Statistical analysis

All experimental results are presented as mean ± standard deviation (Mean ± SD). Data analysis was performed using GraphPad Prism software, with one-way or two-way analysis of variance (ANOVA) used to assess significant differences between treatment groups. P-value of < 0.05 was considered statistically significant.

## Data Availability

The datasets used during the current study are available within the article. The 16S rRNA gene sequence data of zebrafish has been submitted to the NCBI genome database under accession number PRJNA1145643.
